# From “academic success” to “commercial success” —The model of medical device translation driven by SCI articles

**DOI:** 10.1016/j.jot.2025.03.016

**Published:** 2025-04-29

**Authors:** Cheng-Kung Cheng, Chengyan Lin, Yichao Luan

**Affiliations:** a School of Biomedical Engineering, Shanghai Jiao Tong University, Shanghai 200030, China; b Engineering Research Center of Digital Medicine and Clinical Translation, Ministry of Education, Shanghai 200030, China; c Beijing Naton Medical Research Institute, Beijing 100094, China

**Keywords:** Innovative medical devices translation, Unmet clinical needs, Mature medical manufacturing, Project manager, Key opinion leader

## Abstract

For Asia–Pacific countries used to long-term medical device manufacturing, developing innovative medical devices must be rooted in our profound research strength and mature medical productivity. Here, we developed a new model for achieving efficient translation through cooperation among local hospitals, universities, and industries and introduced the project manager system and Key Opinion Leader training. This “From SCI to FDA” model is a pivot for transferring cutting-edge research into valuable medical devices.

**Translational potential of this article:**

This article presents a novel model for translating scientific research into innovative medical devices by fostering collaboration among hospitals, universities, and industries. By integrating clinical insights with academic expertise and industrial capabilities, this model addresses unmet clinical needs and bridges the gap between research and commercialization. Its potential lies in accelerating the translation of cutting-edge research into officially approved products, enhancing medical device innovation, and improving healthcare outcomes globally, particularly in Asia–Pacific countries.

## Graphical abstract

**Figure:**
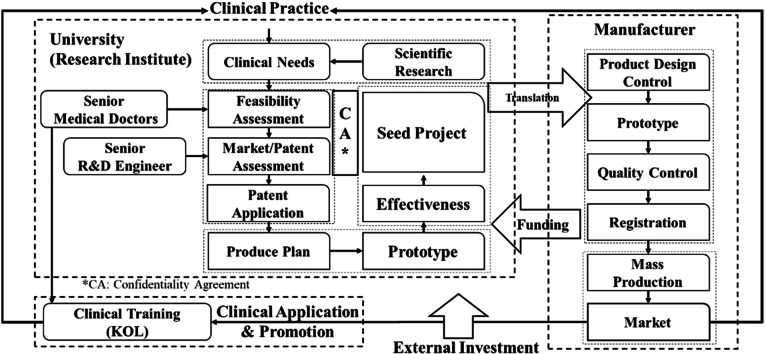

## 1 Introduction

### 1.1 Traditional medical device development model driven by large companies

The research and development of medical devices has always been a “money game." From 2020 to 2023, the total investment and financing of the global healthcare industry was approximately US$331.8 billion. In the first quarter of 2024, it received US$30.7 billion of funding [1]. This preference comes from the more attractive huge commercial market behind. The global medical device market reached US$518.4 billion in 2023 and is projected to exceed US$540 billion in 2024. Among them, medical device spending in orthopedics, cardiovascular, dental, and neuromodulation have been in the leading position [2]. Any successful commercialization of innovative medical device products will bring huge returns, especially in wearable devices and medical AI. The market has witnessed their rapid development in recent years [3]. However, limited by the reality of the medical industry, the translational research of innovative medical devices often takes a long time. Conducting a Class III clinical trial usually requires millions of dollars and takes more than 6 months to years to apply for a registration certificate. Once an accident occurs in any part of the procedure, or the experiment cannot be conducted as scheduled due to a shortage of funds, the setback will lead to rapid failure. We may think the translation research of innovative medical devices is “high risk with high reward."

Global medical device giants dominate the innovative medical device translation market by closely tracking cutting-edge research and market trends. They invest in promising opportunities, quickly acquire or merge with potential innovators, and effectively market these products globally, creating a near-monopoly. This trend is especially prominent in the U.S., where these companies frequently fund university research platforms to secure exclusive advancements.

### 1.2 Thoroughly examining the shortcomings of Asia–Pacific

If we want to figure out our path for innovative medical device translation, we must first start with clearly distinguishing our shortcomings and unique advantages compared to the United States. When considering shortcomings, in addition to the lack of giant medical companies, the doctor training model in Asia generally lacks the “pre-med & post-doctor” mode. For medical students in the United States, before entering medical school, they usually study physics, chemistry, engineering, and even computer science during their four years of undergraduate, then receive clinical training after having specific scientific research capabilities cultivated by undergraduate education [4].

Meanwhile, regarding the hospital's operation and management model, American medical centers usually have a basic research laboratory in each department, employ full-time researchers to conduct independent experiments, and then share results with clinical teams. This practice reduces research pressure on clinicians. The lack of in-hospital laboratories is also a shortage for hospitals outside the United States.

Asian medical device manufacturers have long overlooked innovative research from doctors, opting instead to become original equipment manufacturers (OEM) or imitators for global giants, saving on Research&Development (R&D) and marketing costs. While U.S. companies leverage doctors as global “opinion leaders” to promote new products, their Asian peers rely on sales teams to highlight product similarities. This lack of doctoral involvement in innovation and promotion has led to limited R&D, stagnant industrial upgrades, and products confined to local markets.

### 1.3 Our unique advantages

Everything has two sides, which is also true in our medical device industry. Because of its large population, Asia has become a “key market” of American medical companies. In Asian countries, especially China, Western companies have set up several production bases through self-construction or acquisition, providing convenience for Asian market expansion. In this process, many advanced management experiences and quality control systems have taken root in the local area with the help of foreign medical companies, which also trigger the development of other local competitors. In particular, the ISO 13485 standard, which ensures uniform specifications, safety, and reliability of medical devices, has been widely accepted and used in Asian countries. Regarding production capacity and quality control systems, our manufacturers can meet the strict standards of Western countries. According to the latest medical device industry report, the total amount of medical devices exported by China to the United States in 2023 accounted for 23.18 % of the total amount imported by the United States [5]. This number demonstrates supply chain's high standards and production capacity in China. In recent years, Southeast Asian countries have gradually taken over some of the manufacturing capacity transferred from China for their lower labor costs. In the process, these countries' production and quality control systems have also been greatly improved.

In addition, it is inaccurate to say that Asia–Pacific “does not have sufficient innovation capabilities.” If we look at the number of scientific research articles published, China, Japan, South Korea, and Australia speak loudly. The number of SCI papers published in the Asia–Pacific region is far beyond that of the United States. China's published SCI papers have surpassed that of the United States since 2021, becoming the world's number one. In 2022, China's SCI publications reached 735,600, accounting for 28.9 % of the world's total [6]. However, the low conversion rate from scientific research papers to products is a widespread problem in Asia–Pacific countries. Researchers regard “paper publication” as the end point of research. They do not care about how to commercialize the preliminary results that have been achieved, nor do they understand how to translate. In addition, as mentioned above, we lack large medical companies with strong financial strength and strategic vision like American companies. What is more regrettable is that most of these studies are written in English. After being published in foreign magazines, their research value was discovered by many American companies, becoming their patented products, and even further sold back to the country where the research originated.

### 1.4 Break down the cognitive barriers between academic, medical, and industrial

High-quality research exists globally, with significant potential rewards even if only 1 % of SCI articles are commercialized. While the manufacturing supply chain is ready, the critical issue is that clinicians and researchers have not leveraged it to translate original research. Meanwhile, the industry struggles to find breakthroughs due to a lack of understanding of clinical needs, insufficient research capabilities, and limited experience in promoting original products. To address this, we aim to develop innovative medical devices by bridging academia, medicine, and industry, breaking down cognitive barriers between these fields.

## 2 Our method: from SCI to FDA

### 2.1 Carrying out original research based on “unmet clinical needs”

When a company plans to develop new products, setting up a relatively independent business unit (BU) is an effective method. Building a " BU” with the help of external forces will significantly reduce the company's burden and accelerate the translational process of innovative medical devices. A critical section of BU that can meet the needs is the scientific research expertise of doctors from clinics and universities. Next, we will elaborate on the unique roles of clinics, universities, and manufacturers in innovative translation and how to promote cooperation among the three parties to maximize innovation efficiency.

Innovative medical devices must address “unmet clinical needs” — the foundation of innovation. Clinician involvement is essential to mitigate market failure risks, particularly in the Asia–Pacific region. China's 1.4 billion population generates diverse medical demands, which clinicians distill into actionable needs through continuous clinical experience. However, clinicians should still prioritize patient care over solution development. This helps with cultivating their core value of identifying needs containing clinical and commercial potential.

Once needs are defined, universities and research institutes conduct targeted studies via interdisciplinary collaboration. Academic settings offer advanced equipment and expertise, enabling rigorous validation of safety and efficacy through peer-reviewed publications — a critical step toward translation.

### 2.2 Scrutiny of the project's commercial potential

**Fig. 1:**
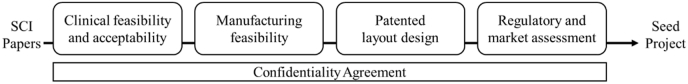
Four-stage evaluation for commercialization of innovative scientific research results.

So far, the development of innovative medical devices has answered the two key questions of “what” - what are our clinical needs; “why” - why this new method can fulfill our clinical needs. We must work with industry to answer the third question: “how” to transform SCI articles into medical devices. However, before actual production, it is necessary to screen through research results for those truly market-worthy ones as the “seeds” for commercialization. At this moment, a third-party evaluation is needed. Under the constraints of the confidentiality agreement, the third-party review needs to be carried out from four aspects (Fig. 1): First, further clinical evaluation, a team of evaluation experts composed of different clinicians systematically analyzes whether the project is indeed a common clinical problem and whether doctors can widely accept the solution proposed in the article; secondly, the manufacturer with cooperating intention analyzes whether the product has the feasibility of large-scale mass production; to protect the intellectual property of the research results, a careful patent layout is necessary; and finally, according to the targeted market, the required route for registration certificate should be designed, and the market plan be evaluated. For the last two points, senior experts who have been deeply involved in the medical device industry for many years can serve as consultants. For start-up teams, we should not only eliminate research that lacks innovation, but more importantly, avoid falling into the trap of high innovation, low returns. Conducting thorough examination among target population, reimbursement landscape, competitive alternatives and revenue model helps reduce the risk of translation.

To achieve efficient cooperation among hospitals, universities, and companies, a structured framework must be established. First, clear agreements on intellectual property (IP) ownership and revenue sharing must be drafted at the outset to prevent disputes. For example, the IP was jointly owned, with royalties distributed based on each party's contribution. Second, regular communication channels (e.g., monthly progress meetings and shared project management platforms) should be established to maintain transparency and address issues promptly. This model not only accelerates the translation process but also fosters long-term partnerships among the participants.

### 2.3 Meeting regulatory requirements for innovative medical devices

Increasing more countries have set up “fast track” for the approval of innovative medical devices. A full understanding of these policies will help innovative teams adjust their strategies and choose the most appropriate certification path, simplifying process and saving time. The following are examples of “fast track” policies set up by some countries (Table 1).

**Table 1:** Innovative medical device approval policies in different countries.

Regulatory agency	Main approval path	Approval cycle (Month)	Initial clinical evidence requirement	Applicable product type
FDA(USA)	510(k) [7]	6–12	Substantial Equivalence	Low to Moderate-Risk
FDA(USA)	Breakthrough Device Program [8]	12–18	Preliminary Clinical Data	High-Risk
NMPA(China)	Innovative Medical Device Special Approval [9]	12–24	Preliminary Clinical Data	High-Risk
PMDA(Japan)	Pioneer Medical Device Designation [10]	12–18	Preliminary Clinical Data	High-Risk
TGA(Australia)	Priority Review Pathway [11]	6–12	Internationally Recognized Data	Moderate-to High-Risk

Meeting regulatory requirements takes time, while making use of these polices can significantly shorten the period, especially in countries like China with stringent approval criteria. During the primary phase, startups should submit innovation plans as early as possible to obtain professional guidance from the review agency. As an example, NMPA may direct companies to leverage local clinical trial sites to generate data that meets its requirements, as international data may not be accepted [12]. In another case, the team may be advised to use foreign-approved medical devices directly in Hainan Special Trade Zone [13], to conduct clinical trial, thereby accelerating the product's certification in China.

Navigating through regulation is a cornerstone of successful medical device translation, particularly for first-in-class innovations. By proactively engaging with regulatory agencies, leveraging fast-track pathways, startups can significantly de-risk their projects and enhance their appeal to investors. Furthermore, a comprehensive understanding of global “fast track” policies enables innovative teams to strategically select the most suitable regulatory pathways [14]. By utilizing the regulatory differences between different countries, we can obtain the goal of “One-time registration, global application”.

Regulatory uncertainties often deter investors from supporting first-in-class innovations, as the approval process can be lengthy and unpredictable. In contrast, me-too products, which leverage existing regulatory precedents, are perceived as lower-risk investments. This preference is particularly pronounced in regions like the Asia–Pacific, where regulatory frameworks are still evolving. To address this challenge, startups must proactively engage with regulatory agencies during the early stages of development to de-risk their projects and attract funding. A clear understanding of these policies is crucial to the project's prospects.

### 2.4 Introduction of project manager system and KOL training

A translational pathway consisting of clinicians, scientific research teams, and manufacturing companies can be contoured in the above process. However, the personnel at each node in this pathway already have their jobs. To maintain the smooth connection between each node, a professional must be involved to supervise the progress of the whole project in real-time; that is, each project needs a full-time project manager (PM). PM plays a pivotal role in ensuring seamless collaboration among hospitals, universities, and companies. Key responsibilities include: (1) timeline management—developing a detailed project schedule and monitoring progress to avoid delays; (2) budget oversight—allocating resources efficiently and ensuring cost-effectiveness; and (3) risk mitigation—identifying potential bottlenecks (e.g., regulatory hurdles or technical challenges) and implementing contingency plans. Project managers do not necessarily need to handle every detail of each project personally. Still, they must have the ability to grasp the direction of the entire team, especially when the project is about to be conducted clinical trials, apply for registration certificates, or face critical financing. In short, the project manager escorts the project's development to always be on the right track. This reduces the pressure and enhances communication among all participants, improving translational efficiency.

By introducing the PM system and combining it with companies possessing production qualifications and capabilities, we answered the three key questions of “what," “why," and “how," and now we have entered the critical stage of product launching and promoting. Learning from the successful experience of large American medical companies, a globally recognized product requires both itself to have enough innovative value and the “key person” responsible for promoting it. The product must be continuously promoted by suitable key opinion leaders (KOL) to gain recognition from users. The person most qualified to undertake this job must and can only be the partnered doctor who has followed the entire process, from discovering “unmet clinical needs” to the product's final launch. They understand the principles and can operate it safely. Manufacturers' original sales system can indeed cope with product promotion to a certain extent, but innovative products differ from OEM products. Only after our KOL doctor does successful surgery can eliminate the concerns of fellow physicians and touch their hearts truly.

However, because of practical limitations, outside the United States, there are insufficient PMs with full-process innovative medical device translation experience, plus clinicians who can take on the KOL's mission. Therefore, training should be thoroughly considered while designing this translational pathway, especially for PMs and clinicians. To achieve this goal, a structured training program must be implemented, focusing on both technical expertise and communication skills. The training methods may contain:

1) Product Knowledge: Detailed technical specifications, clinical applications, and safety profiles of the device.
2) Regulatory Awareness: Familiarity with the approval processes in target markets, including FDA, NMPA, and PMDA requirements.
3) Market Analysis: Understanding the competitive landscape and how to position the device effectively.
4) Ethical Considerations: Guidelines on maintaining objectivity and avoiding conflicts of interest when promoting the device.

Training can be achieved with Seminars, Case-Based Learning, Hands-On Training, together with Communication Skills training. For PMs, the focus of the course is to cultivate their professional qualities, while for KOLs, the focus should be on cultivating their communication and expression skills. These training enable clinicians to complete the career transition from holding scalpels to delivering knowledge. These doctor KOLs will then complete the training of other doctors. By incorporating a systematic training plan into our translational pathway, we can improve the professional capabilities of PMs and clinicians and generate a fission effect through mentorship, promoting the healthy development of the innovative medical device ecosystem in the long run.

### 2.5 Joint shareholding enhances the cohesion of all parts

While the proposed model offers a robust framework for medical device translation, its implementation may face several challenges including resource allocation, conflicts of interest and regulatory hurdles. To address the challenge of maintaining engagement among diverse professionals, the model encourages all participants to jointly invest in a small light-asset technology company, with all participants holding shares together. If there is a need for funds amid project development, social financing can be gradually introduced through equity transfer, especially at the critical start-up stage. Currently, governments provide extensive initial funding support for medical companies. Further understanding of these policies will help companies obtain financing other than venture capital. Successful local government funding plays a more critical role than ever in driving venture capital. Because the participants hold shares together, the enthusiasm of all parties is also strengthened. If necessary, the project manager can also be given a certain amount of equity to enhance his ability to grasp the entire project. When coming to a later stage, the newly established technology company can choose two subsequent development models: Acquisition or IPO. For the first method, the initial investment company has the right to exclusive acquisition, turning it into a real BU. If the initial cooperating company chooses not to acquire, the new company can also seek other potential companies with intentions and production capabilities to negotiate acquisition matters or directly go public and let the market judge its value.

### 2.6 The model that takes advantage of everyone's strength

Our model benefits from the experience of Professor Daifa Wang and his team at Beihang University [15]. Wang's team has made breakthroughs in rapid and accurate imaging of brain areas with black hair in Asians using near-infrared functional brain imaging (fNIRS). This device contains more than 100 channels, helps to understand the mechanism of brain and treat diseases such as stroke and Alzheimer's disease. Based on the research results, Professor Wang and his partners jointly established an innovative company, and through the application of innovative medical projects, achieved rapid registration within two years (in 2019). During translation, Wang's team received millions of yuan in financial support and fee reductions from Danyang City, Jiangsu Province, and his product was also rated as the first major equipment in Jiangsu Province.

In the operation of the company, the position of “Director of Technical Support Department” was set up. Director, Mr. Liang [16], coordinated technical support, research and development, intellectual property rights, pre-sales and after-sales, and put forward key opinions on product improvement in practice. At the same time, their company completes about 30 academic lectures across the country every year, actively promoting the application of fNIRS, which has been promoted to more than 1000 scientific research institutions and hospitals in China. In 2023, Professor Wang's team hosted 20 training sessions in the field of brain science, with more than 8000 doctors trained. In addition, Wang's team also participated in the formulation of China's first mandatory national standard for near-infrared functional brain imaging. Today, they are developing innovative products for the use of fNIRS to treat Alzheimer's disease, also planning to achieve rapid approval through the NMPA medical device green channel.

**Fig. 2:**
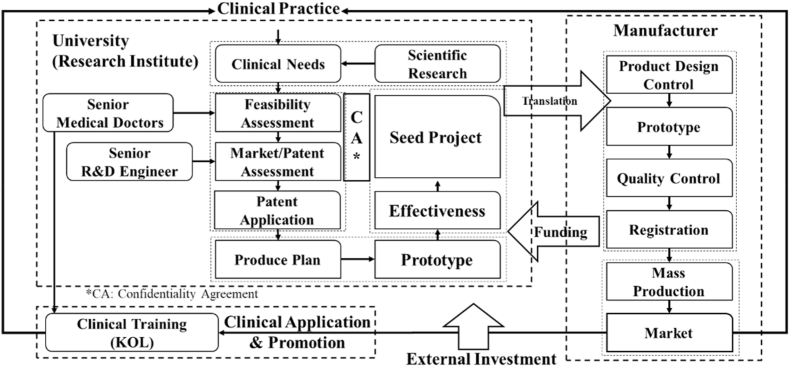
Total solutions for innovative medical devices: from “SCI” to “FDA”.

With Wang's success, we hope to summarize the key points that can benefit all researchers, especially for young researchers who want to achieve translation but do not yet have the mature conditions. From the generation of unmet needs to the sales of products, doctors, researchers, PMs, and companies jointly form a highly efficient converter. The fundamental reason for this model's advantages is that there is no shortage of senior clinicians, high-level SCI articles, and companies with production capacity and qualifications in Asia–Pacific countries. Essentially, we modularize each part of this team, so that every participant can flexibly combine with other cooperative teams at different times according to their own needs, to simultaneously promote diverse translational research. If there is a suitable link to firmly bond these key nodes together, there is an opportunity for innovative medical devices. More importantly, during the project's gradual implementation, the PM will provide opinions from clinical, legal, investment, and industry companions. Meanwhile, projects that have been strictly screened and evaluated are also more likely to be favored by capital. In addition, the existing production capacity may not initially digest the needs of manufacturing new products, but this is also an essential opportunity for industrial upgrading. Our new model solves the problems of initial project sources, mid-term product manufacturing, and final marketing and promotion. It transforms the low-efficient idea of “companies looking for innovative solutions” into the higher one of “innovative ideas looking for productivity," achieving the total solution from “SCI “to “FDA” (Fig. 2).

## 3 Summary

In the future, with the continued expansion of the global healthcare industry, Asia has unignorable potential to become an essential source of international medical device innovation. It is urgent to strengthen cooperation between the clinical, scientific research, and industry communities to achieve this goal. Reinforce the participant with global vision, cultivate a group of PMs with experience in the whole-process management of innovative medical devices, and doctors who can confidently express themselves on international platforms as KOLs. Through in-depth cross-disciplinary and cross-industry cooperation, we can continuously promote the innovation and prosperity of the global healthcare industry. Although innovation is not easy, and the process is full of twists and turns, as long as we master the correct development model and fully develop the potential of local advantages through integration, we can shape a unique path for innovative medical device translation of our own.

## Declaration of AI and AI-assisted technologies in the writing process

No use of any AI and AI-assisted technologies in the writing process.

## Funding

This work was supported by the Fundamental Research Funds for the Central Universities of China (AF0820060).

## Declaration of competing interest

The author has no conflicts of interest to disclose in relation to this article.
